# Automated conserved non-coding sequence (CNS) discovery reveals differences in gene content and promoter evolution among grasses

**DOI:** 10.3389/fpls.2013.00170

**Published:** 2013-07-02

**Authors:** Gina Turco, James C. Schnable, Brent Pedersen, Michael Freeling

**Affiliations:** Department of Plant and Microbial Biology, University of CaliforniaBerkeley, CA, USA

**Keywords:** conserved non-coding sequences, comparative genomics, sorghum, rice, maize, gene regulation, genome evolution

## Abstract

Conserved non-coding sequences (CNS) are islands of non-coding sequence that, like protein coding exons, show less divergence in sequence between related species than functionless DNA. Several CNSs have been demonstrated experimentally to function as cis-regulatory regions. However, the specific functions of most CNSs remain unknown. Previous searches for CNS in plants have either anchored on exons and only identified nearby sequences or required years of painstaking manual annotation. Here we present an open source tool that can accurately identify CNSs between any two related species with sequenced genomes, including both those immediately adjacent to exons and distal sequences separated by >12 kb of non-coding sequence. We have used this tool to characterize new motifs, associate CNSs with additional functions, and identify previously undetected genes encoding RNA and protein in the genomes of five grass species. We provide a list of 15,363 orthologous CNSs conserved across all grasses tested. We were also able to identify regulatory sequences present in the common ancestor of grasses that have been lost in one or more extant grass lineages. Lists of orthologous gene pairs and associated CNSs are provided for reference inbred lines of arabidopsis, *Japonica* rice, foxtail millet, sorghum, brachypodium, and maize.

## Introduction

Conserved non-coding sequences (CNSs) are islands of non-coding sequence that show an unexpectedly low level of divergence. In plants these sequences are identified by comparison of non-coding regions surrounding homologous genes. The ideal window to identify the CNS most likely to have biological function is to compare genomic regions which have experienced between 0.5 and 0.9 synonymous substitutions per site (Freeling and Subramaniam, 2009). For less diverged homologous genomic regions, some functionless sequences will still retain detectable sequence similarity, while in more diverged genomic regions many functionally constrained sequences will have diverged too much from each other to be identified as homologous, with only the largest, most conserved CNSs remaining detectable. While many CNS are expected to function as cis-regulatory regions involved in regulating transcription and chromatin structure, the specific function of most plant CNSs remains unknown (Freeling and Subramaniam, 2009). As with mammals (Loots et al., 2000), there are several cases in plants of CNSs being proved to contain functioning cis-regulatory regions, as reviewed (Freeling and Subramaniam, 2009) and (Raatz et al., 2011). An early genome-wide analysis of CNSs in plants focused on duplicate genes in arabidopsis (*Arabidopsis thaliana, At*) resulting from an ancient whole genome duplication (Thomas et al., 2007). Such retained pairs of genes are called homeologs, or homoeologs, Ohnologs or syntenic paralogs. Regulatory genes tend to be associated with larger quantities of these CNSs than are other classes of genes and these CNSs are significantly enriched in transcription factor binding motifs. The G-box and G-box-like sequences were the most enriched in CNSs as compared to all other known transcription factor binding sites or random 7-mer motifs (Freeling et al., 2007). Recent work in rice has shown a postive correlation between open chromatin and CNSs (Zhang et al., 2012). Arabidopsis homeologs with many associated five prime CNS tend to show less expression than arabidopsis genes with fewer CNS (Spangler et al., 2012). There is also evidence that genes with the most associated CNS (CNS-richness) are more likely to be retained following whole genome duplication, perhaps because of selection against disruption of DNA-protein stoichiometries (Schnable et al., 2011) or perhaps because they are more readily subfunctionalized (Force et al., 1999).

Plant genes are generally associated with shorter and fewer CNSs than mammalian genes at similar divergence (Inada et al., 2003) and are expected to degrade relatively quickly in comparison to mammalian CNSs (Reineke et al., 2011). The most studied plant CNSs are a set of 14,944 CNSs identified through the examination of 6,358 homeologous gene alpha (retained from the most recent tetraploidy) pairs in arabidopsis (Freeling et al., 2007; Thomas et al., 2007), based upon an updated list of those first identified by Bowers and coworkers in the Patterson lab (Bowers et al., 2003). The process of manually proofing each CNS took two people two years of effort and the resulting large set of sequences provides a standard against which automated methods can be compared. The automated CNS Discovery Pipeline was developed to replicate the logic and consistency checks performed by a human proofer, and compensates for many of the complexities of both annotation and biology which were identified as problematic by human proofers, including errors in gene structure, clusters of locally duplicated homologous genes, gaps in the pseudomolecule assembly, repetitive sequences and similar sequences at non-syntenic locations relative to the anchoring homologous gene pair.

**Figure 1:**
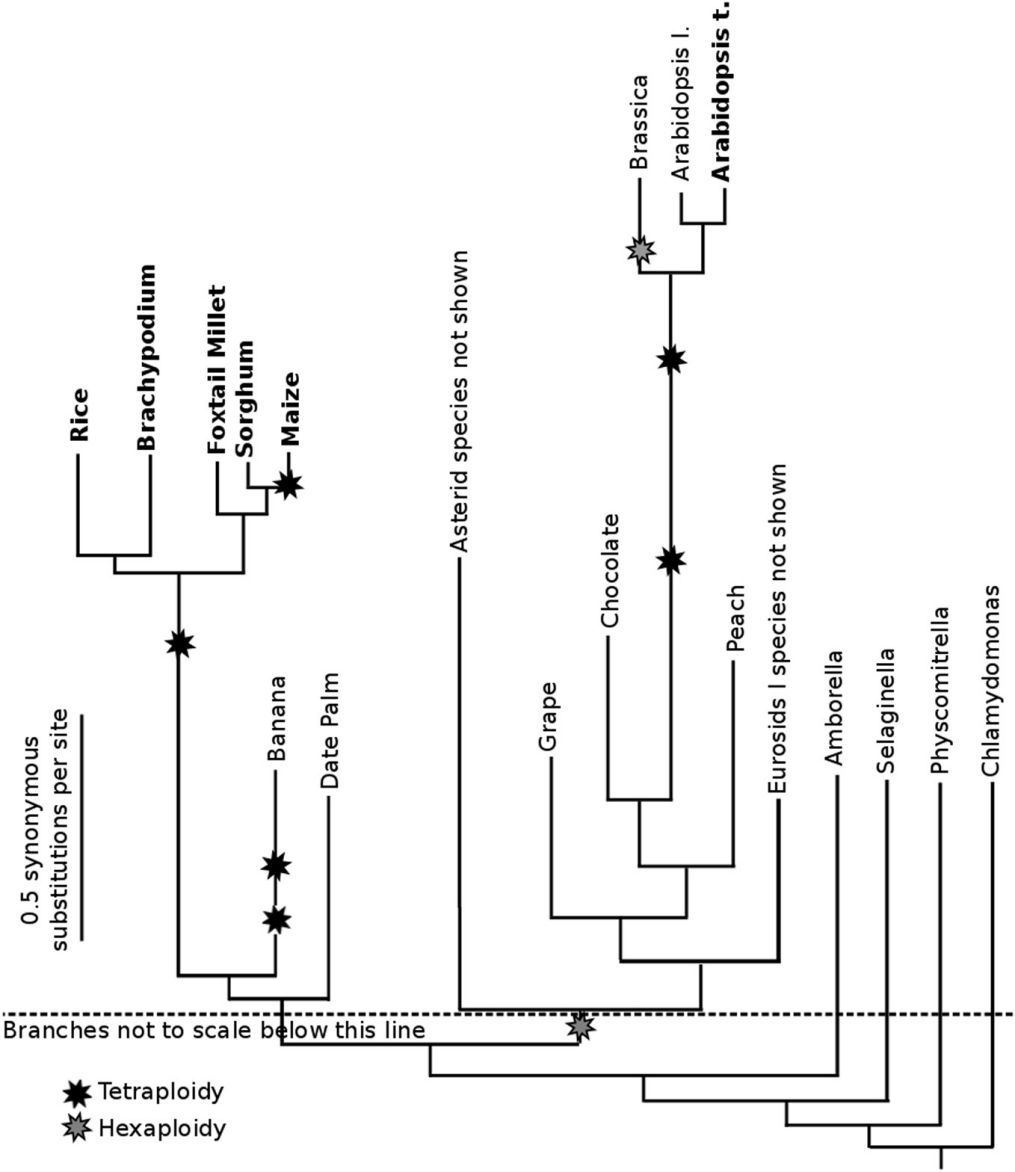
**Phylogenetic relationships among published plant whole genomes.** Branch lengths are scaled based on the modal number of synonymous substitutions per nucleotide (modal Ks) between the protein coding open-reading-frame of orthologous (or homeologous) genes. The total Ks separating orthologous genes in two species can be assessed looking at the total length of all branch segments traversed to connect the two species names. The total Ks separating homeologs in a given species is the distance from the species name to the starburst representing that whole genome duplication and back to the species name. Unequal branch lengths from a common ancestor indicate the acceleration on base pair substitutions in certain lineages. Each boldfaced species is involved in one or more pairwise data sets, the output of the CNS Discovery Pipeline 3.0: Supplemental Data Sets use the suffix “a” for orthologous or homeologous syntenic gene lists, and the suffix “b” for the CNS list. They also use two letter abbreviations for genomes based on species names. For example, *Oryza sativa = Os*. Syntenic orthologs and Ks values were calculated using SynMap (Lyons et al., 2008).

The whole genome duplication which occurred in the ancestor of all grasses (Paterson et al., 2004), as diagrammed in Figure 1, also occurred within the useful window of pairwise CNS discovery (modal synonymous substitutions per site 0.5–0.9; in this case 1.0 remains useful). For that reason, comparing the genes on the subgenomes of grasses is useful for CNS discovery. In addition, we identified CNSs by comparing orthologous genes between different pairs of diverged grass species. As seen in Figure 1, rice-sorghum and rice-setaria are ideally diverged for CNS discovery. Few difference would be predicted between these sister orthologous gene lists and sister CNS lists. Comparing the genomes of sorghum and setaria directly is informative, but these panicoid grasses are too closely related; CNSs discovered using our standard significance cutoff (equal to or more significant than a 15/15 exact match) would include those carried-over even though they had no function.

Plant genomes sequenced to date (Figure 1) are skewed toward species with smaller, more compact genomes. Orthologous CNSs were identified between rice and maize (*Zea mays, Zm*) to test the pipeline under the more challenging conditions presented by the average plant. The recently sequenced maize genome (Schnable et al., 2009) has increased 2.5-fold in size relative to sorghum, its close sequenced relative (Figure 1), as the result of multiple transposon blooms (Baucom et al., 2009) and a whole genome duplication (Gaut and Doebley, 1997) 5–12 million years ago (Swigonova et al., 2004). This whole genome duplication means the modern maize genome consists of two duplicate subgenomes, each potentially containing an ortholog for any gene shared with other grass species. Both duplicate genes (Woodhouse et al., 2010; Schnable et al., 2011) and duplicate CNSs in maize are fractionating (fractionation refers to the loss of duplicate sequences following whole genome duplication), so the tetraploidy certainly introduces additional complexity.

## Results

### Accuracy of automated CNS identification

**Figure 2:**
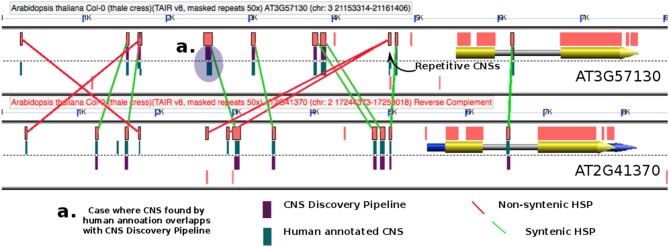
**Exemplary GEvo panel (Lyons and Freeling, 2008) depicting BlastN HSPs (orange rectangles) resulting from on-the-fly comparison of one particular homeologous gene pair in *Arabidopsis thaliana*.** Coding exons are yellow while non-coding UTRs are blue. The TAIR8 genomes being used by GEvo is decorated by drawing purple rectangles for CNSs discovered by our Pipeline 3.0 (customized genome = dsid19494 available as a pull-down option in GEvo “Sequence Submission”) and green rectangles for CNS discovered manually. Previously identified CNSs are displayed by selecting the “show pre-annotated CNSs” option in GEvo. Illustrated are manual red lines for “CNSs” that are non-syntenic, and in masked regions and manual green lines for syntenic CNSs. Regenerate these data in GEvo http://genomevolution.org/r/8ooz (Set reverse complement to achieve this orientation).

The accuracy of the pipeline was gauged by comparing the *At-At* homeologous CNS previously identified by manual annotation (Thomas et al., 2007; Subramaniam et al., 2013) to those identified through the CNS Discovery Pipeline 3.0 (Figure 2, Table S1 and Supplemental Data Sets 1 and 2). When the coordinates of a manually annotated CNS overlapped with the coordinates of a CNS discovered by the pipeline the CNS was scored as correctly identified (e.g., Figure 2). Eighty percent of the manually annotated CNSs were identified by the CNS Discovery Pipeline (Table S1). Re-examination of the CNSs found uniquely in the manually annotated data set revealed that 54% were non-syntenic relative to other CNSs occupying the same gene space, a standard that was less stringently enforced in the generation of that dataset. The CNS Discovery Pipeline also does not consider low complexity CNSs (like ATATATATATATATAT; 14% of manual annotation-specific CNSs) and does not examine complex repetitive sequences present at 50 or more locations in the genome (22% of manual-annotation specific CNSs, see Methods). The remaining 10% of manual-annotation specific CNSs (198 CNSs) was removed by an additional filtering step performed by the CNS Discovery Pipeline which was not feasible for human annotators: CNSs showing sequence similarity to any annotated coding sequence (CDS) elsewhere in the genome or possessing significant, putative RNA secondary structure are considered possible unannotated RNA or protein-coding genes, not CNSs. The CNS Discovery Pipeline also identified 1,777 CNSs (out of a total of 12,088) that were missed by manual annotation. Almost all of these were located more than 4 kb (kilobases) away from the anchor gene or within introns. Based on these comparisons the overall accuracy of the CNS Discovery Pipeline appears to be greater than manual annotation of CNSs in arabidopsis-arabidopsis comparisons.

Figure 2 demonstrates how manually annotated CNSs and the CNS Discovery Pipeline 3.0 CNSs were compared using the GEvo graphical display. GEvo is a sequence comparison tool and an application in the CoGe comparative genomics toolbox (Lyons and Freeling, 2008). We used a CoGe customized (decorated) TAIR8 genome, dsid 19494, to visualize our pipeline CNSs as purple rectangles on the model track (Figure 2). Each row of CNS data included in Supplementary Data Sets online includes a link to GEvo allowing researchers to generate outputs similar to Figure 2 for any CNS or gene pair. Note in Figure 2 that the typical pipeline CNS (purple) matches a manually annotated CNS (green) and is verified by BlastN data, as depicted in the exemplary case identified in Figure 2 as “a”.

### Applying the CNS discovery pipeline to find orthologous CNSs in new species

The CNS Discovery Pipeline 3.0 was used to identify CNSs in the grasses. This aim required the identification of syntenic orthologs between *Japonica* rice (*Os*) and sorghum *(Sb*) and independently between rice (*Os*) and setaria (*Si*). Both sorghum and setaria are panicoid grasses, a clade which is estimated to have diverged from rice around 50 million years ago (Kellogg, 2001). The modal synonymous substitution rates (Ks) between rice-sorghum orthologs and rice-setaria orthologs are quite similar (modal Ks between syntenic orthologous genes were 0.56 and 0.55 substitutions per site, respectively) as expected given their shared divergence event. This modal Ks is within the window of useful CNS discovery (Freeling and Subramaniam, 2009). We reasoned that comparisons of sorghum and setaria to the rice genome should show similar patterns of conservation. Syntenic blocks were identified using the QUOTA-ALIGN algorithm (Tang et al., 2011). Overall, the rice-sorghum comparison has fewer and smaller syntenic blocks than the rice-setaria comparison. This difference may be a byproduct of differences in genome size, sequence coverage, and in assembly methods employed in the two species. Note that all three species are diploid relative to each other, although all three share a common whole genome duplication that occurred in the ancestor of all grasses (Figure 1).

Table 1 compares the conservation of protein CDS between sorghum and rice to the conservation between setaria and rice (comparison of pipeline Gene Lists: Supplemental Data Sets 3A and 4A). Rice genes without a syntenic ortholog but with a homologous gene identified by LASTZ (Harris, 2007) at a non-syntenic location are labeled as a “best hit,” and these genes are not assumed to be orthologous. Clusters of local duplicate genes are collapsed and treated as a single gene. While the total number of homologous genes between rice and sorghum and between rice and setaria are similar, the numbers of these genes at syntenic locations (orthologs) differ significantly (Table 1).

**Table 1:** **Gene conservation in *Os-Sb* and *Os-Si***.

**Gene category**	**Sorghum**	**Setaria**
Total officially annotated genes (MSU6 *Japonica* rice = 57624)	33996	35853
Rice gene with a syntenic ortholog	16251	17210
Rice genes without a syntenic ortholog but a hit with an e-value <1e-10 to a non-syntenic gene	12343	11345
Total conserved rice genes	28594	28555
Number of lineage specific (not shared; *Sb* or *Si* only) genes losses	988	1067
Number of lineage specific (not shared with *Os*) genes	2204	5632

In addition to total gene conservation, the pipeline-derived data set was used to determine individual gene loss or gain from a syntenic location. Each rice gene with an ortholog present in one species (sorghum or setaria) but not the other was labeled as lost in the corresponding species. A gene is recognized as gained if no ortholog is present in rice, brachypodium (like rice, a member of the BEP grass clade), and setaria (for candidate lineage specific genes in sorghum) or sorghum (for candidate lineage specific genes in setaria). Table 1 shows that, by these criteria, the setaria genome has gained and also has lost more genes than sorghum. GO (http://www.geneontology.org) annotations for these genes were compared to annotations for all genes in a Fisher Exact Test using the Bonferroni method to correct for multiple testing. Genes gained in sorghum are enriched in annotations related to “transposons” and in genes with no functional annotation. Setaria-gained genes are also significantly enriched in the above two terms, with the addition of “drought induced.”

In addition to showing greater numbers of genes conserved at syntenic locations, setaria also shows higher levels of non-coding sequence conservation—relative to the rice genome—than observed in the sorghum genome. Table 2 compares the CNS data sets produced by the CNS Discovery Pipeline 3.0 for rice-sorghum and rice-setaria (Supplemental Data Sets 3B and 4B, respectively). Approximately 10,000 fewer CNSs were identified in the rice-sorghum comparison than the rice-setaria comparison. This effect was observed independently of differences in the number of syntenic gene pairs identified in the two comparisons as individual gene pairs tended to have both more and larger CNS identified between rice and setaria than between rice and sorghum (Table 2). Note that the two comparisons share a common absolute divergence date as sorghum and setaria shared a common ancestor more recently than their shared divergence from the lineage leading to rice (Figure 1).

**Table 2:** **Summary of CNS distributions in *Os-Sb* and *Os-Si***.

**CNS data**	**Sorghum**	**Setaria**
Total number of (orthologous^1^) CNSs	52958	64466
% of orthologs^1^ with at least 1 rice CNS	79.00%	80.00%
Average number of rice CNSs/pair	3.15 CNS/gene	3.61 CNS/gene
No. of Bigfoot genes (large gene spaces)^2^	767 genes	949 genes
Mean length of rice CNSs	34.78 base pairs	36.87 base pairs
Median length of rice CNSs	26 base pairs	27 base pairs
Total quantity of conserved non-coding sequence	1.84 megabases	2.38 megabases
% of CNS 5′ distal	19.92%	20.06%
% of CNS 5′ proximal^3^	14.29%	13.31%
% of CNS 5′UTR	10.36%	9.99%
% of CNS intron	21.49%	23.38%
% of CNS 3′ UTR	14.132%	14.11%
% of CNS 3′ Proximal	7.27%	7.01%
% of CNS 3′ distal	12.54%	12.14%

1 Gene information includes “CNSs” reassigned as orthologous RNA genes or protein-coding exons.

2 Genes were identified as Bigfoot if the total non-coding space between CNSs, or between the furthest CNS and exon, was at least 4 kb. Each Bigfoot gene must also have at least one CNS every 1 kb.

3 Proximal regions were identified as any region located 1 kb from the start or end of the transcription unit.

To further investigate this unexpected difference between lineages, the setaria CNS sequence (>30 bp (base pairs) derived from comparing *Os-Si* and which were “unique” to rice-setaria gene pairs) were used to probe the gene space surrounding orthologous sorghum genes. While setaria and sorghum are too closely related to rule out neutral carryover as an explanation for similar sequences, this comparison makes it possible to track the fate of CNSs identified only in rice-setaria comparisons and undetectable in rice-sorghum comparisons. Of the 41% of CNS “unique” to rice and setaria and greater than 30 bp long, 50% can be identified surrounding orthologous genes in sorghum when using the setaria CNS sequence as a probe. This suggests rice-setaria CNS are not deleted in sorghum but instead have diverged sufficiently in sequence to be undetectable in comparisons to rice. If studies of gene loss in maize and *Brassica rapa* are representative of the fate of functionless DNA in all plants, then functionless DNA is quickly deleted in plants rather than slowly randomized by base pair substitutions (Subramaniam et al., 2013). Thirty-eight random 5′ distal, *Os-Si* CNSs greater than 30 bp unidentified in *Os-Sb* were examined manually in GEvo panels using various alignment algorithms and settings. None of the 38 were deleted in *Sb*. 37 of 38 were present in *Sb*, but BlastN hits fell just below our defined CNS cutoff. These CNSs were detected by decreasing our BlastN cutoff from an e-value <15/15 exact match to <13/13 exact match. One CNS was not found because it was too far away and filtered out through the bowtie algorithm of the pipeline; only this one of 38 constitutes a pipeline “error.” The loss/turnover of some CNSs during evolution is challenging and these follow-up experiments suggest that the lineage-specific CNS loss seen here is not the result of CNS deletion, as will be discussed.

**Figure A1:**
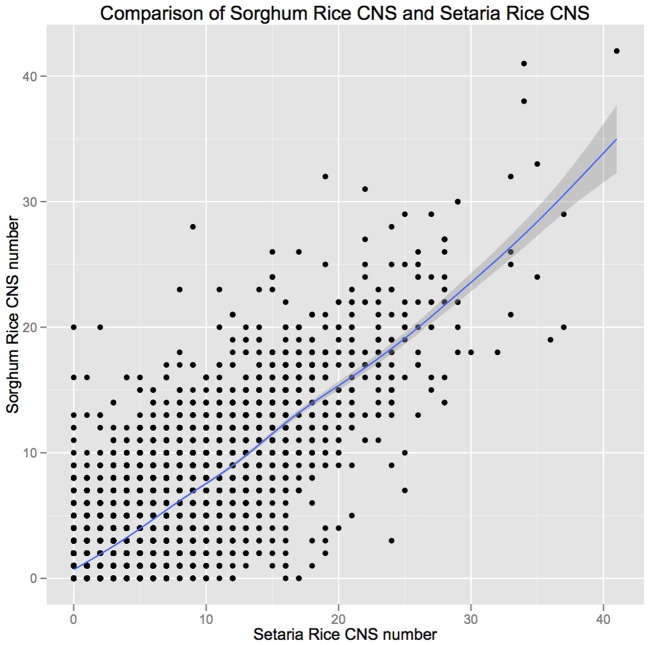
**Figure A1. Comparison of the number of discrete conserved noncoding sequences discovered for orthologous sorghum and setaria genes when compared to their common ortholog in rice**.

The overall distribution of CNSs relative to their genes (five prime, intronic, three prime) was equivalent in both comparisons, with a ratio of roughly 1.3:0.6:1 of five prime:intronic:three prime positions (Table 2). This enrichment of 5′ CNSs is lower than was previously reported for homeologous CNSs in arabidopsis (Thomas et al., 2007). Note the homeologous arabidopsis CNSs are considerably more diverged than are the orthologous CNSs (Figure 1). For rice genes with syntenic orthologs in both sorghum and setaria, the number of CNS identified in each pairwise comparison was also significantly correlated (Pearsen's *R*^2^ = 0.82, Figure A1). The most CNS-rich gene in comparisons to both setaria and sorghum is *Os03g20090*, a MYB family transcription factor gene.

### Handling unusually large and unusually small genomes

The maize genome is repetitive, large, and abundant in transposons, providing a difficult environment for identification of CNS. To compensate for maize's large size and large number of non-syntenic genes, more relaxed parameters were used for the identification of syntenic regions. While this relaxation makes it more likely false syntenic regions and syntenic regions dating from ancient whole genome duplications will also be introduced, these contaminating syntenic blocks are removed during the quota-filtering step of QUOTA-ALIGN. While the search space used for identifying [query (--qpad) and subject (--spad)] was kept at the default of 15 kb up and downstream for rice, it was increased to 30 kb in maize. It was also necessary to implement a new “large_genome” option in the CNS Discovery Pipeline. This option allows greater differences between species in the spacing of a CNS relative to its associated gene in large transposon rich genomes such as maize where nests of transposon insertions can drastically change the spacing of promoter elements. The “large_genome” option also triggers an additional step to attempt to correct for cases where contigs generated by sequencing of bacterial artificial chromosomes were placed onto pseudomolecules in the wrong order or orientation (Schnable and Freeling, 2011) by identifying synteny between CNS within individual contigs. This option should become more useful as more large and difficult-to-assemble genomes are sequenced, such a barley (International Barley Sequencing Consortium, 2012) and wheat. Allowing users to adjust these features provides a highly versatile program for identification of CNS, although this large genome option does affect the comparability of CNS whole-genome data. Roughly 60,000 rice CNSs were identified from the *Os-Zm* comparison with the settings adjusted to search a large complex gene space (Supplementary Data Sets 5A and 5B). The maize genome is not too large or ambiguously assembled for CNS discovery, however, large genomes do require modifying the criteria initially developed for working in the compact genomes of model species.

Identifying CNS in large and small genomes represent two fundamentally different challenges. As the genome gets smaller, genes are packed closer together and it becomes more difficult to accurately identify the correct gene to assign a CNS. The approach of the CNS Discovery Pipeline takes into account the distance to the nearest conserved gene pair up and downstream of the gene in both genomes being compared. The test case for small genome size was *Brachypodium*, the smallest grass genome sequenced to date (270 mb). In a comparison of the rice and brachypodium genomes 70,000 orthologous CNSs were identified (CNS Discovery Pipeline 3.0 ran without the large genome option; Supplementary Data Sets 6A and B).

### Pan-grass CNSs

The CNS identified in rice-sorghum, rice-setaria, and rice-brachypodium comparisons were combined using the genome coordinates of the CNS in rice. This resulted in a set of 15,363 CNSs that were identified in all three analyses (Supplemental Data Set 7). These “well behaved” CNSs can be considered to be under the most stable purifying selection and appear to not be affected by binding site turnover (Venkataram and Fay, 2010), switching among “dormant” binding sites (Junion et al., 2012) or any other scheme that shuffles functional sites among redundant sites. In addition, these stable conserved sequences double the number of syntenic anchor sequences to aid in syntenic path assembly of genomes from additional grass species (Mayer et al., 2011), and aid in developing genetic maps and mapping mutants and quantitative traits for species without significant genomic resources.

### Intragenomic pairs and homeologous (alpha) CNSs

Pairs of genes retained from a whole genome duplication are called homeologs (Syn. homoeologs, syntenic paralogs, Ohnologs, alpha paralogs, in-paralogs). Because whole genome duplications duplicate all regulatory sequences along with the genes these sequences are associated with, CNS can be identified between homeologous genes, as was done for the arabidopsis–arabidopsis CNSs (Supplemental Information 1 and 2). Rice, brachypodium, sorghum, and setaria are all descended from a tetraploid ancestor and homeologous genes in each species are within the useful window for CNS discovery (Figure 1). We have prepared the Pipeline 3.0 homeologous Gene List (suffix a) and homeologous CNS List (suffix b) for three grass genomes as Supplemental Datasets 8A and B to 10A and B, respectively. A cursory examination found much similarity between these different datasets, as expected if the majority of promoter fractionation occurred in the time between the pre-grass whole genome duplication and the divergence of the major grass lineages, as was observed for the fractionation of whole genes in these lineages (Schnable et al., 2012).

### Biological utility example 1: enrichment of the label “transcription factor” and particular go terms in orthologous grass CNSs as compared to non-CNS non-coding control sequence

**Figure 3:**
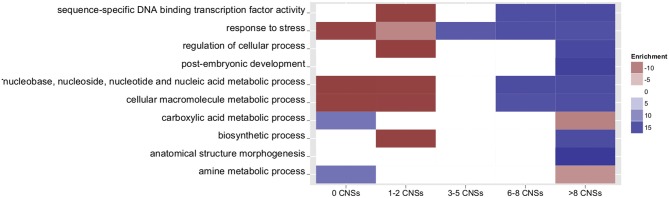
**Number of associated Os-Si CNSs and GO term enrichment for selected go terms.** Only terms with a corrected *p*-value of ≤ 0.001 are counted as over- or under-represented. White blocks denote insignificant enrichment values. Colors indicate fold enrichment.

Several studies have shown that regulatory genes tend to be associated with greater numbers of CNS in plants, as reviewed (Freeling and Subramaniam, 2009). To assess whether orthologous grass CNSs follow a similar pattern, genes were grouped based on number of associated rice-sorghum CNSs and gene ontology (GO) terms were compared among groups (Figure 3, a complete list of enriched GO terms is provided in Table S2). Genes with many CNSs are enriched in the annotations related to “development” and “response to” (GO:0032502 and GO:0050896). Genes with fewer CNSs tend to be metabolic and housekeeping genes. This finding is generally consistent with previous observations of arabidopsis homeologous CNSs (Thomas et al., 2007). This finding agrees with the model that genes expressed at constant levels throughout development and under all environmental conditions utilize less complex regulation than genes whose expression changes between cell types, tissue/organ types, developmental time points, or external conditions. *Os-Si* CNSs are also similar to arabidopsis in the association of genes encoding transcription factors with CNS-richness: 48% of rice-setaria genes with at least 25 CNSs encode transcription factors while only 4.6% of pairs with 0 CNS encode transcription factors. Similar results were obtained for rice-sorghum comparisons. Individual CNSs associated with genes encoding transcription factors are also more likely to have been identified in both rice-sorghum and rice-setaria comparisons.

### Biological utility example 2: g-boxes and other DNA-protein binding motifs in orthologous CNSs and their possible link to drought stress

Many CNSs are binding sites for transcription factors. Thus, it is expected that the CNS sequences will be enriched in known functional binding motifs. For the homeologous CNSs of arabidopsis, the most enriched motifs were the G-box motif, and G-box like sequences (Freeling et al., 2007). To determine enrichment of these motifs a regular expression was used to find all non-overlapping matches within CNSs and control sequences selected from the promoters of the same grass genes (see Methods). Rice-sorghum CNSs were enriched 2.5X in the extended G-box (5′ACGTGGC), 2.3X for the G-box, and 3.8X for the telo-box (5′AAACCCTAA) relative to a control set of non-conserved non-coding sequences. To identify additional enriched motifs in grass CNSs, CNS were compared to an equivalent set of non-conserved non-coding control sequences using DREME (Bailey, 2011). Motifs enriched in CNS were compared to the 469 published cis-acting regulatory elements contained within the PLACE database (Higo et al., 1999). Over 60% of motifs significantly enriched in CNS correspond to at least one PLACE motif (Table S3). Many of these enriched and characterized motifs were identified as being involved in various “response to” pathways. The two most significantly enriched motifs were the G-box (CACGTG) and the MYCATERD1 box (5′CATGTG) which is implicated in early response to dehydration (Tran et al., 2004). Other enriched motifs are listed in and our complete DREME data set in Table S3.

**Figure 4:**
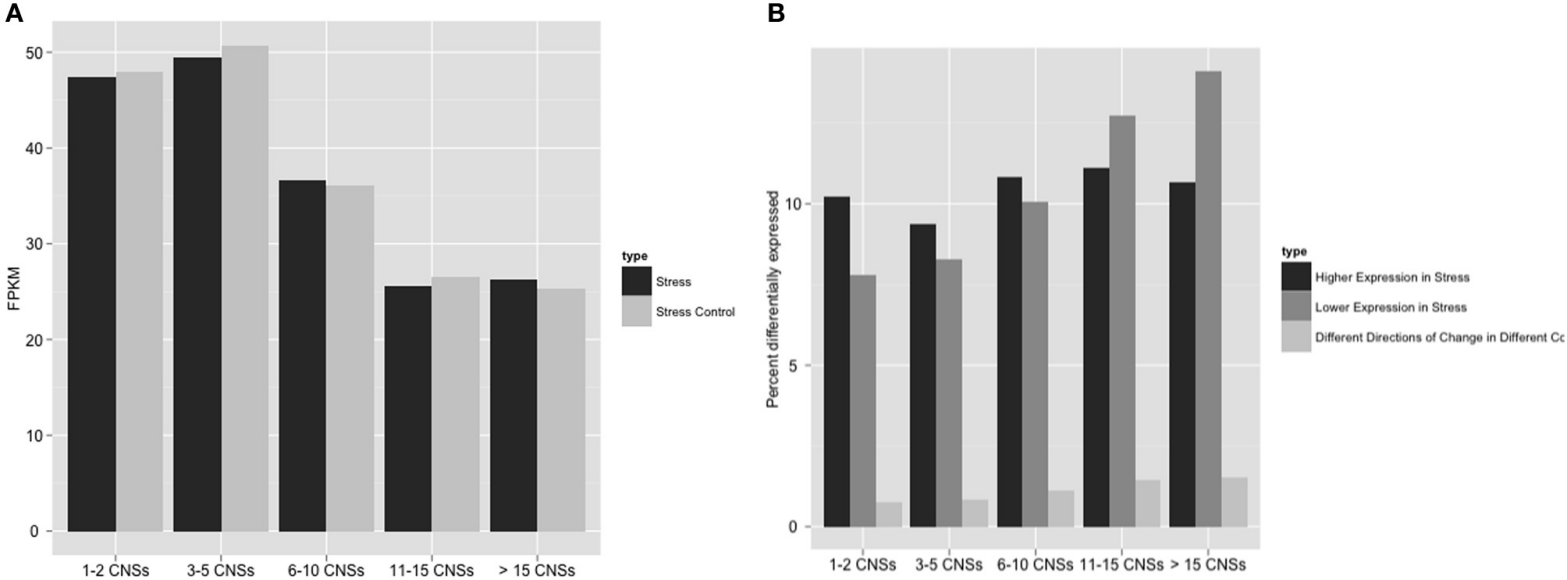
**Number of *Os-Sb* CNSs and association with both raw expression levels and differential expression in sorghum.** Panel **(A)** compares the average raw expression values for stress and stress control to CNS richness of genes. “Stress” here are FPKMs in response to PEG + ABA treatment (Dugas et al., 2011). **(B)** Differentially expressed categories, as defined exactly in Methods, display the percent of the genes differentially expressed in each group. “Differential expression” is a measure of difference between induced and control, so the most dramatic data above is the increasing *down regulation* (negative) of stressed gene expression as the number of CNSs increase.

To more directly investigate the link between CNS richness and stress response, we took advantage of an existing stress response RNA-Seq dataset in sorghum. The Klein lab characterized changes in the expression of sorghum seedling shoots and seedling roots in response to the hormone ABA and simulated osmotic stress produced by the application of polyethylene glycol (Dugas et al., 2011). Using the RNA-Seq reads generated in that set of experiments we found that genes with many CNS tended to have globally lower expression levels regardless of environmental conditions (Figure 4), consistent with a previous microarray-based study of the link between conserved promoter complexity and gene expression in arabidopsis (Spangler et al., 2012). Genes with few CNS were equally likely to show up or down regulation when sorghum seedlings where exposed to ABA or osmotic stress. However, genes in the highest categories of CNS richness tended to show even lower expression in ABA treated or osmotically stressed seedlings than seedling grown under controlled conditions (Figure 4). CNS associated with differentially expressed genes were enriched in ABA related motifs, particularly the ABA responsive element (ABRE: 5′ACGTGG) (Narusaka et al., 2003) and ABASEED core elements (5′ACGTGC), which respond to ABA only in developing seeds (Thomas, 1993).

## Discussion

### Promoter and *cis*-regulatory annotation in the age of abundant sequenced genomes

The CNS Discovery Pipeline 3.0 was applied in pairwise fashion to multiple genomes. The pipeline is able to largely replicate the results of manual annotation of CNS, and requires approximately 30 min of one programmer's time as opposed to the efforts of two trained biologists over a two-year period. As whole genome sequencing becomes increasingly commonplace, many tools have emerged for the rapid and automatic annotation of protein coding exons. Yet transcribed sequence is only a portion of the gene. To truly understand a gene it is important to also characterize the regulatory sequences that determine when and where the protein a gene encodes will be produced. Despite immense progress/advancement in the field of comparative genomics, there are still a very limited number of tools for CNS identification, particularly in plants where non-coding sequence diverges at much higher rates than observed in animals. Our pipeline represents one approach to identifying potential functional regulatory sequence in an automated and high-throughput manner. The pipeline also provides an unbiased platform for CNS discovery. Past human annotation turned out to be biased toward 5′ CNS assignments relative to the gene rather than 3′. This could explain some of the distribution discrepancies found between manual annotation and the pipeline. Additionally the pipeline further increases accuracy by using known RNA and protein sequences to filter likely transcribed sequences that have thus-far escaped annotation. Thus, our pipeline is not only useful for discovery of CNS but for identifying protein coding genes, RNA-genes, and pseudogenes that had not been annotated previously. Finally, our pipeline has the same chance of finding a CNS 12 kb upstream from the nearest CDS, as it does in the proximal promoter. This is important for discovery of distant enhancers and similar elements known to function in animal systems (Bulger and Groudine, 2010).

### Comparison with other automated methods of conserved non-coding sequence discovery

Baxter et al. (2012) identified thousands of arabidopsis and other dicot CNSs by comparing promoter regions globally among four sequenced dicots: *Arabidopsis thaliana*, papaya, poplar and grape and demonstrate the functional relevance of their CNS through correlations between CNS-richness and regulatory GO terms, known DNA-binding motifs and nucleosome occupancy data. They state, correctly that their moving 60 bp window method—the “seaweed” method—of find an optimized global alignment over repeatmasked, paired queries is more sensitive than the blastn, the local alignment algorithm used by PL3. The cost of this sensitivity is that the seaweed approach is only practical for the discovery of CNS located within the proximal non-coding region—specifically the 2 kb region upstream of the TSS. PL3, on the other hand, uses Blastn to find local HSPs anywhere in a large window including all of the gene and all sequences to the next paired gene, skipping over transposons and out-of-synteny genes in the process without penalty. 3′, intronic and very distant CNSs are detected if syntenic and above 29.5 bitscore (the e-value of a 15/15 exact match). For example, the 5 kb of CNS rich space 5′ to the arabidopsis genes of Figure 2—gene encoding a adaxial-abaxial axis protein-binding function—would have been largely excluded by the methods of Baxter and coworkers only because most of these CNSs are too far distal of the TSS. Combining CNS identified using multiple techniques is likely the best approach to identify exhaustive sets of conserved functional non-coding sequences.

### Unequal genomic structure divergence between sister panicoids sorghum and setaria

The growing wealth of genome assemblies now available in the angiosperms empowers researchers to move beyond simply identifying conserved sequences between two species. It is now possible to compare CNSs identified among multiple species allowing identification of conserved sequences present in a common ancestor but deleted from the genomes of one or more descendant species. In this study we compared the genomes of two panicoid grasses, setaria, and sorghum, to an outgroup species, rice (Figure 1). Since setaria and sorghum share a common divergence from the lineage leading to rice and show similar rates of synonymous substitutions between orthologous genes, the two pairwise comparisons were expected to reveal generally similar patterns of conservation in both coding and non-coding sequence. Contrary to that expectation, setaria shows both a larger number of syntenically conserved genes and more/larger CNSs associated with each gene. Since the rate of base substitution in the two lineages is the same, the difference must be caused differences in the rate of some courser mutagenic mechanism, like indels or small intrachromosomal recombination-type deletions (Hollister et al., 2010). Or, perhaps insertions cause the erosion of CNS detectability. The genome of sorghum is larger and more repeat-rich than that of setaria. It has been argued that sorghum's large transposon-rich pericentromeric regions are evidence of large transposon blooms in the history of that lineage (Paterson et al., 2004). The disruptive effects of both transposon insertion and deletion may be responsible for at least some differences in conservation we observed between the two genomes. Transposons can contribute to the erosion of synteny, by serving as potential recombination sites for inversions and translocations (Montgomery et al., 1991) and by capturing host genes and inserting them at non-syntenic locations within the genome (Jiang et al., 2004; Brunner et al., 2005). These differences and rearrangements in CDS in sorghum could also account for differences in the number of CNSs identified between the two pairwise comparisons. It is also possible that a sudden increase in DNA content, by polyploidy, as with some fish lineages (Lee et al., 2011) induces mechanisms that tend to reduce DNA content.

Use of CNSs identified between sorghum and setaria in comparison to CNSs “unique” to one species (rice-setaria or rice-sorghum) turned out to be an effective way to detect grass CNSs that are real but on the boarder of detectability. Fifty percentage of CNSs “unique” to only rice and setaria (not detected in rice-sorghum comparisons) are detected in sorghum by using orthologous setaria CNS sequence to probe the gene space. This result indicates that many functionally constrained sites do not consistently show enough sequence conservation to rise above the threshold of detectability. A large number of functionally constrained sites, which are sometimes above, and sometimes below the threshold of detectability explains why the number of CNS associated with orthologous genes in sorghum and setaria is highly correlated despite the fact that many individual CNS show no overlap between the two species.

### Traits correlated with promoter size

Grass genes with large conserved promoter regions are different from other genes, as they tend to be “regulatory” (Inada et al., 2003). Even in the absence of data on functional sequences within promoters, it has been demonstrated that arabidopsis genes with large five-prime non-coding regions separating them from the next upstream gene show more complex patterns of expression in response to external stimuli (Sun et al., 2010). A previous study of arabidopsis homeologs determined that Bigfoot genes (genes with large gene spaces and many CNSs) are generally enriched in GO terms related to “transcription factor” and “response to” while genes with few CNSs are associated with household and/or metabolic GO terms (Freeling et al., 2007). As CNSs were previously only identified between arabidopsis genes with retained homeologs, these correlations were determined using only a subset of the genome. Genes with retained homeologs are already biased toward certain functional GO categories (Blanc and Wolfe, 2004; Seoighe and Gehring, 2004; Maere et al., 2005). The current set of orthologous CNS data allowed the study of a population of genes more representative of the total gene set of plant species. The broader representation of gene types provided by comparisons between orthologous genes lead to the discovery that genes associated with regulation of development are also over represented among the most CNS rich grass genes. Some examples include: “post-embryonic development” and “anatomical structure morphogenesis.” Genes with few CNS are expected to be involved in “housekeeping” with consistent patterns of expression (Thomas et al., 2007). It should be noted that the annotation “cellular macromolecule metabolic process” GO term was enriched among CNS rich genes, but these same genes tend to also be annotated as “regulation of.” This is consistent with the hypothesis that CNS-richness correlates positively with a more complex pattern of expression.

While functional annotations provide a broad view of gene function, RNA-Seq experiments now make it possible to identify specific differences in the expression patterns of CNS-rich and CNS-poor genes. The fact that CNS-rich sorghum genes were more likely to show differential expression in response to stress was consistent with the results of GO analysis. However, GO analysis alone would not have revealed the fact that this pattern was only present when examining genes that showed lower expression in response to environmental stimuli. This suggests that the average CNS rich gene may function in pathways sensitive to changes in the external environment, rather than directly regulating the responses of a plant to changes in its environment.

The experimentally determined function of CNS rich genes also supports this hypothesis that genes associated with many CNS tend to be those that must be expressed only at specific times and/or places. For example, in our data set of homeologous rice CNSs from the pregrass whole genome duplication, the most CNS rich gene is OS06G40780. This gene is better known in rice as MONOCULM1 (Li et al., 2003), it is expressed only within developing axillary meristems, and is involved in the control of rice tillering. Uncharacterized grass genes with large complements of CNSs are likely to also perform crucial functions in plant development or environmental response and represent promising targets for future genetic characterization.

Genomes are composed largely of non-protein-coding DNA. Identification of CNSs in plants provides a method for separating the rare functional non-coding sequence from the vast majority of zero-function or low-function sequence within the genome. Having a subset of non-coding DNA “known to function” should generally advance our progress toward discovering the function of individual sequences and understanding the language of gene expression regulation. Our pan-grass CNS list organized on the orthologous pan-grass genes (Supplemental Data Set 7) provides this subset of known functional elements for the grass family. Since these pan-grass CNSs are about as conserved as CDS sequences and show more syntenic conservation than the average gene, they should serve as useful anchors for the assembly of additional genomes based on conserved synteny, in translating map positions from sequenced to unsequenced grass species, and in more accurate genetic mapping.

## Conclusion

The source code for our CNS Discovery Pipeline 3.0 is freely available for download (https://github.com/gturco/find_cns) and handles both the identification of syntenic orthologs or homeologs using the previously published algorithm QUOTA-ALIGN (Tang et al., 2011) and the identification, proofing, and gene pair assignment of CNSs. To facilitate proofing of our results and further experimentation, direct links to CoGe comparisons are provided in each row of our many “gene list” output data spreadsheets (Supplemental Data Sets 1A–6A and 10A–12A). The CNS Discovery Pipeline 3.0 should be generally useful for improving gene annotations and CNS discovery when usefully diverged genomes are available, including those of species suffering from transposon blooms or massive chromosomal rearrangements. Although the pipeline was designed to accommodate the multiple whole genome duplications characterizing angiosperms, it functions equally well on genomes throughout the tree of life.

With the increasing number of sequenced plant genomes becoming available, particularly in the grasses and crucifers, there is great potential for phylogenetic footprinting to inform both our understanding of conserved gene regulation and also to identify specific loss of individual cis-acting regulatory modules in specific lineages. Global alignments of genomes of multiple species anchored on exonic sequences will certainly generate more accurate phylogenetic footprints when close to conserved exonic anchors (Baxter et al., 2012; Haudry et al., in review). However, this pipeline excels in finding functionally constrained sequences located multiple kb away from the nearest conserved feature such as distal enhancers or repressors of gene expression. The genetic code was cracked over a half-century ago. A combination of different approaches will ultimately be needed to finally decode the language of gene regulation.

## Methods

### Pipeline 3.0

**Figure 5:**
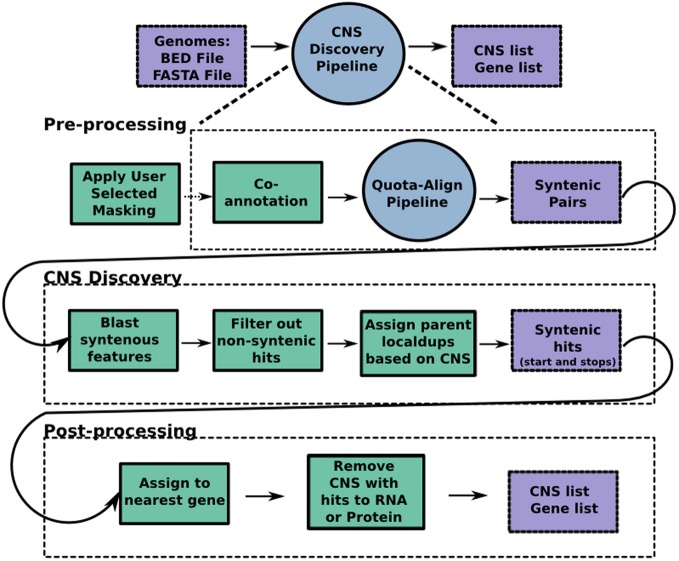
**The CNS Discovery Pipeline 3.0.** The pipeline can be divided into three stages: pre-processing, CNS discovery, and post-processing. Co-annotation, where each genome helps find missed genes in the other, occurs during preprocessing, so any new genes found become available potential syntenic gene spaces for CNS discovery. Purple boxes represent input and output files while green boxes represent python scripts that make up each program. Each circle represents an individual program, the CNS Discovery Pipeline being the largest of the programs. The primary script for the QUOTA-ALIGN pipeline is published (Tang et al., 2011). Masking genomes is computationally expensive and time consuming. Researchers can download many pre-50× masked genomes from CoGe (masked using the script referenced in methods) or apply their own masking tools to screen out repetitive and low complexity sequences.

The source code for our CNS Discovery Pipeline 3.0 is available for download at https://github.com/gturco/find_cns with instructions for installation at (https://github.com/gturco/find_cns/blob/master/INSTALL.rst). Running the pipeline requires the genomic sequence in FASTA format and annotation data in BED format for each genome being compared. The CNS Discovery Pipeline produces two data sets per run, the gene list (suffix “a” in our Supplementary Data Sets) and the CNS list (suffix “b”). For each gene in the genome, the gene list reports any identified syntelog, CNSs and local duplicates. Proofing early versions of the automated output of the CNS pipeline was conducted using GOBE visualization software (Pedersen et al., 2011). The CNS list reports detailed information on each individual CNS identified by the pipeline: DNA sequence, location in the genome, associated gene (usually closest), and position relative to that gene. A CNS list can be loaded into CoGe (Lyons and Freeling, 2008) upon request. Pipelines 2.0 and 3.0 were proofed in GEvo the sequence alignment application of CoGe using genomes with CNS annotations. The following steps, summarized in Figure 5, were automated through the use of python, perl and UNIX scripts included within the CNS Discovery Pipeline.

### Preparing genomic sequences

Sequences were masked for any repeats that occurred over 50 times in the entire genome of each species using a self-self-blast of the entire genome. BlastN was ran using a word size of 15 bp (−W 15) with an e-value < 0.001 (−e 0.001). An “N” replaced any base-pair position covered by 50 or more separate blast hits. The scripts used for this step is available from http://code.google.com/p/bpbio/source/browse/trunk/scripts/mask_genome/mask_genome.py Masked repetitive sequences are color-coded pink when a genomic region is displayed by the CoGe application GEvo.

To avoid errors in analysis, which can result from genes missed by the official annotation of a genome, fasta files were re-annotated through comparison of the query and subject sequence. We refer to this process as co-annotation. Sequences were compared using BlastN, at a word size of 20 bp (−W 20) and an e-value cutoff of 0.001 (−e 0.001). When a single gene showed similarity to multiple regions within the genome and were separated by less than one kb, these hits were merged into a single co-annotated gene. If the total length of merged similar regions was less than 100 bp or blast hits covered less than 40% of the total length (start of the 5′ most blast hit of the merged group to the end of the 3′ most hit) the region was discarded. If the region was located within an already annotated gene, it was assigned to that gene as a missed exon(s). Regions in intergenic space were considered to represent either missed genes or unannotated pseudogenes and added to our in-house annotations of the genome using the naming convention: organism_chromosome_start_stop_strand (these annotations are provided in the, pipeline output, Supplemental Data Sets for each species). These new CDS found by co-annotation are color-coded purple when viewed in GEvo if the genome selected contains “with CNS PL3.0” in the title line. Note that co-annotation provides new genes/pseudogenes that may or may not prove to be syntenic with other genes, and thus may or may not provide a new gene space for CNS discovery. Table S3 provides a list of our customized genomes in CoGe, with their unique identification numbers.

### Finding syntenous regions

The CDS of each official and newly annotated gene in the query and subject genomes were compared using LASTZ (Harris, 2007) run with default parameters. The results were filtered with the Blast_to_raw script from the quota alignment package [https://github.com/tanghaibao/quota-alignment; (Tang et al., 2011)]. Homologous genes located near each other in the genome, separated by no more than twenty intervening genes (--tandem_Nmax 20), were clustered into a single group when their similarity score was greater than 0.5 (--cscore 0.5). After CNS discovery was performed (see below) only the gene copy with the most CNS was retained for further analysis. The filtered data was used to locate the appropriate orthologous or homeologous syntenic blocks with QUOTA-ALIGN (Tang et al., 2011) using a 20 gene distance cutoff for extending the chain (--Dm 20) a 4 gene minimum chain size (--min_size) and quotas appropriate to each comparison; users have the option of changing these setting before running the pipeline. For example, comparing *Os* to *Zm* uses a quota of 1:2.

### Finding CNS between syntenous regions

For each syntenic gene pair identified by QUOTA-ALIGN, regions of sequence starting 12 kb upstream of the annotated start site of each gene and extending 12 kb past the end of transcription were extracted from the 50× masked genomic sequence files. In addition to the 50× repetitive sequence masking all annotated protein coding regions (CDSs) were also masked. Bl2Seq was used to compare the two regions using the following parameters: wordsize 7 bp (-W 7), gap penalties extension 2 (-E2), nucleotide mismatch penalty 2 (-q 2), nucleotide match reward 1 (-r 1), cost to open a gap 5 (-G 5), and DUST filtered turned “on” (-F T). Hits with a bitscore less than 29.5 [equivalent to a perfect match of 15 base pairs (Kaplinsky et al., 2002)] were discarded.

### Filtering out non-syntenic blast hits

Any blast hit not present in the same orientation relative to the syntenic gene pair was discarded. The remaining potential CNSs were treated as two-dimensional objects using the geographic library GEOS (http://trac.osgeo.org/geos/) with python bindings provided by Shapely (http://toblerity.github.com/shapely/index.html). Using the intersection function of Shapely, any potential CNSs located in the intron of one pair but not the other was also removed. If a potential CNS overlapped with another potential CNS, the potential CNS with the least significant e-value was removed iteratively until no overlapping CNSs remained. Potential CNSs in non-syntenic locations were also removed if they crossed over three or more other potential CNSs. If any of the remaining CNSs were still in conflicting syntenic relationships, the conflicting CNS with the lowest bitscores were iteratively removed until all remaining CNSs were present in the same order in both genomes. To further enforce synteny, a two dimensional expanding polygon shaped like a bow-tie with the midpoint of each gene at the center was created through GEOS and Shapely. All potential CNS outside this polygon were discarded. The bow-tie shape of the polygon confirms that the position of one CNS relative to its associated syntenic gene is similar to the position of the corresponding CNS and its syntenic gene. Increasing discrepancies in position were tolerated further upstream/downstream from the respective gene. CNSs falling within the polygon were found using a point in polygon route. (http://www.ecse.rpi.edu/Homepages/wrf/Research/Short_Notes/pnpoly.html). Practically speaking, this bow-tie confirms synteny between homologous CNSs, within 12 kb of the start and end of any paired genes.

### Filtering out CNSs with hits to arabidopsis proteins and RNA

All CNSs > 18 bp in length were filtered by comparison to all arabidopsis TAIR10 proteins CNS with a LASTZ hit to arabidopsis protein at an *e*-value < 0.01 and >90% coverage were re-annotated as a missed gene/gene fragment and discarded. CNS were also compared to annotated non-coding RNAs within Arabidopsis TAIR10. BlastN was run at a wordsize of seven bp (-W 7) and at an *e*-value cutoff of 0.001 (-e 0.001). CNSs with hits to annotated RNAs were re-annotated as RNA and discarded.

### Assigning CNS to genes

CNSs were assigned to genes based on the nearest syntenous feature. When the same CNS was identified in the comparison of multiple syntenic genepairs, the genepair to which it is assigned is determined by two rules. First, the CNS is assigned to the gene pair with fewer intervening non-syntenic genes (up to a maximum of three). When there were no intervening non-syntenic genes or equal numbers up and downstream of the CNS, the CNS was assigned to the gene pair separated from the CNS by the smaller number of total base pairs.

The location of each CNS was classified as intron, five prime or three prime UTR (untranslated region), five prime or three prime proximal, or five prime or three prime distal. A CNS is considered to be in a UTR if it overlaps with an annotated UTR exon of either member of the syntenic gene pair. A CNS is identified as proximal if it is located <1 kb from the start or end of the transcription unit, and distal if it is located further away from the gene. Genes were classified as “Bigfoot” if the gene pair was associated with at least four CNS spread over a non-coding 5′ + 3′ region of at least 4 kb and with at least one CNS every 1 kb of non-coding space.

### Pipeline graphic output: customized CoGe genome

CNSs, RNAs, and unannotated genes identified by the pipeline were loaded into the CoGe database for visualization in GEvo. Genomes annotated with these additional features are marked by a PL2 or PL3 in their name, depending of the CNS pipeline version. PL2 and PL3 differ only by a small change in how we deal with tandem repeat genes. A data set identification number (dsid) denote a genome in CoGe that is available in GEvo using the pull-down menu. To view CNSs click “Show pre-annotated CNSs” under Results Visualization in GEvo. *Os* dsid 47668, for example, contains both *Os-Sb* CNSs and *Os-Si* CNSs denoted as colored rectangles on opposing strands. This genome is available from a pull-down menu in GEvo when rice is used as either the subject or query in any genome. Upon clicking a CNS in GEvo the annotation will appear indicating on which two organisms and genomes the pipeline was run. For example, at the time of this paper's publication, there were a total of 15 different arabidopsis (*At*) genomes available in CoGe, comprising different TAIR releases plus several different customizations. Contact coge.genome@gmail.com for genome questions or to load a new customized CoGe genome. For annotated genomes the customization can be exported as GFF or TBL from the “Dataset Information” box, under the “Organism View” application of CoGe, and individual features making up any annotation may be downloaded as a text “type, chromosome, start, stop, strand, length.” A list of customized genomes in GEvo, and information necessary to point to each in a GEvo URL (Uniform Resource Locator) is in Table S3.

### GO term enrichment

All enrichment and purification of GO-terms reported in this paper were calculated using the goatools python package (https://github.com/tanghaibao/goatools). The GO annotation file was retrieved through the MSU Rice Genome Annotation Project (Ouyang et al., 2007). Co-annotated genes identified by the CNS Discovery Pipeline were not included. Enrichment was determined using a Fisher's exact test. The false discovery method was used to correct for multiple testing. Results were considered significant at a *p*-value of < 0.001. Complete GO enrichment data for rice genes by CNSs are in Table S2.

### Measurement of Motif Enrichments

Over-represented motifs were identified by DREME [Discriminative Regular Expression Motif Elicitation (Bailey, 2011)] with the minimum core size set to 6 bp. Each motif found in the CNS fasta file is compared to the non-conserved-non-coding control sequence generated from the non-conserved-non-coding sequence located 15,000 bp up and downstream the gene. Non-conserved-non-coding sequences consist of all genomic sequences excluding CDS, CNS, and masked sequence. The Bonferroni multiple testing correction was applied with a *p*-value cutoff of 0.005. A regular expression was used to report all non-overlapping occurrences of motifs also found in the PLACE database (Higo et al., 1999). Random subsets of CNSs were used as control motifs to calculate the significance of the enrichment of characterized binding sites. A two-tailed chi-squared test was used to determine significance of a motif appearing in PLACE. Table S4 shows all 103 of these significantly CNS-enriched motifs.

### Transcription factor analysis

Transcription factor information was downloaded from the Database of Rice Transcription Factors (Gao et al., 2006) in June, 2012. Genes were matched to TF based on gene name. Enrichment analysis was performed using a two-tailed chi-square test.

### Syntenic hits and best hits

Data sets for rice gene comparisons were obtained from LASTZ and syntenic pipeline outputs (described above). LASTZ results were further filtered for distinct hits with an e-value < 1e-10. Local duplication sets, genes interrupting a local duplicate array were = 3, remained condensed as the pipeline ran. New candidate genes identified by the CNS Discovery Pipeline (co-annotated) were not included. Annotations enriched for lineage specific or in one lineage but not in the other were identified through a Fisher exact test. False discovery rate corrections were used to correct for multiple comparisons. Annotations with a *p*-value above 5% were considered significant.

### Expression data

Data on the expression of sorghum genes in response to stress, used as an example of the biological utility of CNS data, was obtained from Dugas et al. (2011). Using the raw sequence data from the International Nucleotide Sequence Database Collaboration (INSDC) Sequence Read Archive (short reads archive), expression values were calculated for each gene by aligning reads to the sorghum genome using GSNAP (Wu and Nacu, 2010) and calculating expression in units of FPKM using Cufflinks (Roberts et al., 2011). The average expression value for stress was calculated across all three replicates of ABA and PEG. NAOH and H20 were used for calculating the average expression value under unstressed conditions. Lists of genes showing statistically significant differential expression were taken directly from those calculated by Dugas et al. (2011) using the EdgeR statistical software package (Robinson et al., 2010) to analyze digital gene expression data. A gene was considered positively regulated in response to stress if it was significantly up regulated in at least one of four treatment/organ combinations (ABA roots, ABA shoots, PEG roots, PEG shoots) and did not show significant down regulation in any treatment/organ combination. The same logic was used to identify genes negatively regulated in response to stress. Genes classified as “both” were those that showed significant up regulation in at least one treatment/organ combination and significant down regulation in at least one other treatment/organ combination. Table S5 shows the resulting FPKMs.

## Authors' contributions

Gina Turco participated in design, implementation, and analysis of all experiments and drafted the manuscript. Gina Turco also implemented the final version of the CNS Discovery Pipeline 3.0. James C. Schnable participated in design of experiments and interpretation of results, analysis of RNA-Seq data, and helped to draft the manuscript. James C. Schnable also contributed to the design of the pipeline and the large genome parameter of the pipeline. Michael Freeling contributed the conceptual model of the CNS pipeline, and participated in proofing, experimental design, interpretation of results, and helped to draft the manuscript. Brent Pedersen implemented the CNS Discovery pipeline 1.0.

### Conflict of interest statement

The authors declare that the research was conducted in the absence of any commercial or financial relationships that could be construed as a potential conflict of interest.

## Supplementary material

The SupplementaryMaterial for this article can be found online at: http://www.frontiersin.org/Plant_Genetics_and_Genomics/10.3389/fpls.2013.00170/abstract

Table S1 **Table comparing manual CNSs to pipeline output**.

Table S2 **Complete CNS-enriched GO terms**.

Table S3 **CNS-enrichment for select motifs**.

Table S4 **Customized CoGe genomes: dsid and dsgid**.

Table S5 **Our FPKM workup of *Sb* reads from (Dugas et al., 2011)**.

Supplemental Datasets are hosted on figshare: http://dx.doi.org/10.6084/m9.figshare.107054

Supplemental Data Set 1 ***At-At* manually annotated v2 CNS list (with genes)**.

Supplemental Data Set 2A ***At-At* PL2 Gene list**.

Supplemental Data Set 2B ***At-At* PL2 CNS list**.

Supplemental Data Set 3A ***Os-Sb* PL3 Gene list**.

Supplemental Data Set 3B ***Os-Sb* PL3 CNS list**.

Supplemental Data Set 4A ***Os-Si* PL3 Gene list**.

Supplemental Data Set 4B ***Os-Si* PL3 CNS list**.

Supplemental Data Set 5A ***Os-Zm* PL3 gene list**.

Supplemental Data Set 5B ***Os-Zm* PL3 CNS list**.

Supplemental Data Set 6A ***Os-Bd* PL3 Gene list**.

Supplemental Data Set 6B ***Os-Bd* PL3 CNS list**.

Supplemental Data Set 7 **Pangrass (*Os +Bd +Sb +Si*) PL3 CNS list with gene info**.

Supplemental Data Set 8A **Os*-Os* PL3 homeologous Gene list**.

Supplemental Data Set 8B ***Os-Os* PL3 homeologous PL3 homeo**.

Supplemental Data Set 9A ***Sb-Sb* PL3 homeologous Gene list**.

Supplemental Data Set 9B ***Sb-Sb* PL3 homeologous PL3 homeo**.

Supplemental Data Set 10A ***Si-Si* PL3 homeologous Gene list**.

Supplemental Data Set 10B ***Si-Si* PL3 homeologous PL3 homeo**.
